# Charting the cellular landscape of pulmonary arterial hypertension through single-cell omics

**DOI:** 10.1186/s12931-024-02823-0

**Published:** 2024-05-03

**Authors:** Brian Tang, Arjun Vadgama, Bryce Redmann, Jason Hong

**Affiliations:** Division of Pulmonary and Critical Care Medicine, David Geffen School of Medicine at UCLA, University of California, 200 UCLA Medical Plaza, Suite 365-B, Box 951693, Los Angeles, CA 90095 USA

**Keywords:** Pulmonary arterial hypertension, Single-cell RNA sequencing, Single-cell omics

## Abstract

This review examines how single-cell omics technologies, particularly single-cell RNA sequencing (scRNAseq), enhance our understanding of pulmonary arterial hypertension (PAH). PAH is a multifaceted disorder marked by pulmonary vascular remodeling, leading to high morbidity and mortality. The cellular pathobiology of this heterogeneous disease, involving various vascular and non-vascular cell types, is not fully understood. Traditional PAH studies have struggled to resolve the complexity of pathogenic cell populations. scRNAseq offers a refined perspective by detailing cellular diversity within PAH, identifying unique cell subsets, gene networks, and molecular pathways that drive the disease. We discuss significant findings from recent literature, summarizing how scRNAseq has shifted our understanding of PAH in human, rat, and mouse models. This review highlights the insights gained into cellular phenotypes, gene expression patterns, and novel molecular targets, and contemplates the challenges and prospective paths for research. We propose ways in which single-cell omics could inform future research and translational efforts to combat PAH.

## Introduction

**Fig. 1:**
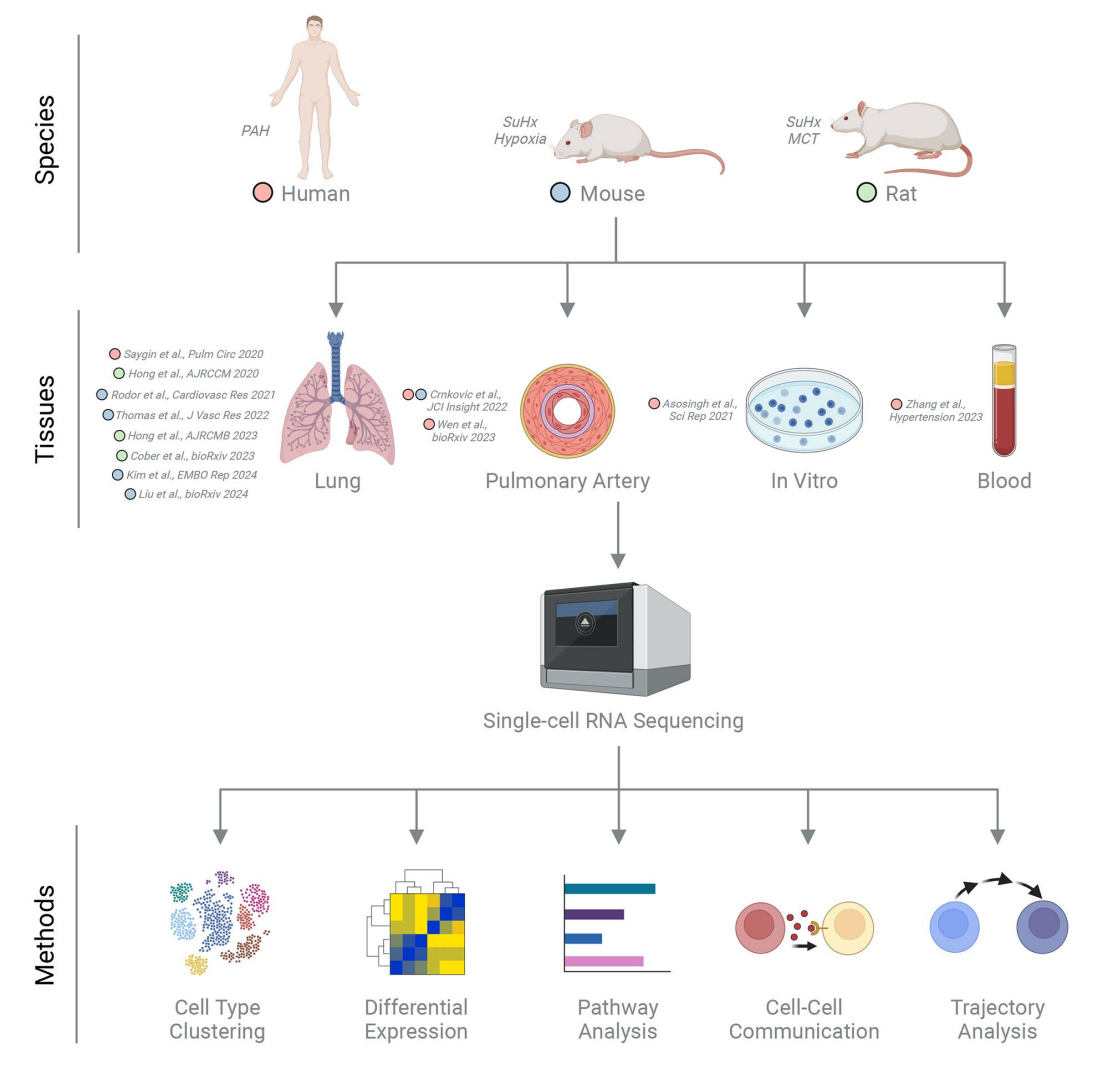
Summary schematic of scRNAseq studies in PAH. Different colored dots indicate which species the study focused on: red = human; blue = mouse; green = rat. Created with BioRender.com. SuHx = Sugen-hypoxia; MCT = monocrotaline.

Single-cell RNA sequencing (scRNAseq) represents a paradigm shift in our ability to investigate the transcriptome at a granular level, unlocking the cellular heterogeneity of tissues in health and disease. This review delves into the transformative impact of scRNAseq and its potential to offer unprecedented insights into the pathobiology of pulmonary arterial hypertension (PAH)—a condition marked by complex vascular remodeling leading to right heart failure and high mortality. Central to this remodeling are vascular cells such as endothelial cells (ECs), which adopt a hyperproliferative and apoptosis-resistant phenotype. In addition, inflammation and immune dysregulation may also drive vascular remodeling in PAH, with various immune cells implicated in disease pathogenesis. The advent of scRNAseq has enabled a detailed look into the transcriptomic landscape of the diverse array of vascular and immune cells that may play a role in PAH pathobiology. Traditional molecular approaches, while foundational, offer a limited view, often silencing the chorus of cellular interactions and heterogeneity fundamental to the disease. This review charts the recent advances enabled by scRNAseq, illuminating key discoveries in diverse cell populations across human, rat, and mouse models of PAH. Following this introduction, we will begin with a discussion of technical considerations for setting up scRNAseq experiments. Our literature search focused on studies published from 2020 to 2024, reflecting the most recent and relevant findings in the field. We synthesize major insights, underscore the challenges faced, and propose directions for future research in single-cell omics that could propel the field forward and advance clinical strategies for PAH. The scRNAseq datasets informing our discussion are summarized in Table 1 and Fig. 1. Our literature retrieval was guided by keywords such as ‘pulmonary arterial hypertension,’ ‘single-cell omics,’ and ‘single-cell RNA sequencing.’ Studies focusing on subgroups of pulmonary hypertension (PH) other than WHO Group 1 PAH, such as chronic thromboembolic pulmonary hypertension (CTEPH), were intentionally excluded from this review. This decision was made to maintain a clear focus on the distinct pathobiological features of WHO Group 1 PAH, which is the primary subject of our analysis and discussion.

**Table 1:** **Summary of scRNAseq datasets in PAH.** Only newly generated datasets are included for a given study. Previously published datasets that were reanalyzed were excluded

Authors	scRNAseq platform	Species	Tissue	Sample size	Cells	PMID	Data availability
Saygin et al., *Pulm Circ* (2020)	10x Genomics Chromium 3’ v1/v2	Human	Lung	IPAH (*n* = 3) Control (*n* = 6)	30,480	32,166,015	GSE169471
Hong et al., *Am J Respir Crit Care Med* (2020)	Drop-Seq	Rat	Lung	SuHx (*n* = 6) MCT (*n* = 6) Control (*n* = 6)	33,392	33,021,809	http://mergeomics.research.idre.ucla.edu/PVDSingleCell/.
Asosingh et al., *Sci Rep* (2021)	10x Genomics Chromium 3’ v2	Human	PAEC	PAH (*n* = 3) Control (*n* = 3)	72,454	34,282,213	GSE185479
Rodor et al., *Cardiovasc Res* (2021)	10x Genomics Chromium 3’ v2	Mouse	Lung	SuHx (*n* = 3) Control (*n* = 3)	27,557	34,528,097	GSE154959
Thomas et al., J Vasc Res (2022)	10x Genomics Chromium 3’ v2	Mouse	Lung	4-week Hypoxia (*n* = 1) Control (*n* = 1) ^‡^	~ 5,000	35,294,950	GSE209738
Crnkovic et al., *JCI Insight* (2022)	10x Genomics Chromium v2	Human (*Mouse)	Pulmonary artery	IPAH (*n* = 3) Control (*n* = 3)	22,704	36,099,047	GSE228644
Zhang et al., *Hypertension* (2023)	BD Rhapsody	Human	Peripheral blood	IPAH (*n* = 3) Control (*n* = 3)	21,315	37,313,754	N/A
Wen et al., bioRxiv (2023)	10x Genomics Chromium 3’ v3.1	Human	Pulmonary artery	PAH (*n* = 3) Control (*n* = 3)	62,375	N/A	N/A
Cober et al., bioRxiv (2023)	10x Genomics Chromium 3’ v3	Rat	Lung	SuHx (*n* = 4) Control (*n* = 4)	~ 25,000	N/A	N/A
Liu et al., bioRxiv (2024)	10x Genomics Chromium 3’ v3	Mouse	Lung (ECs)	CKO, EH2, WT (*n* = 3^†^/group)	6,910	N/A	N/A
Kim et al., EMBO Rep (2024)	10x Genomics Chromium 3’ v3.1	Mouse	Lung (CD45^−^CD326^−^)	3-week Hypoxia (*n* = 3^†^) Control (*n* = 3^†^)	N/A	38,243,138	GSE249461

^†^pooled samples.

^‡^ Chronic hypoxia vs. control experiment was also performed in *Notch3*-Null mice (*n* = 1/group).

IPAH = idiopathic pulmonary arterial hypertension; PAEC = pulmonary artery endothelial cell; SuHx = Sugen-hypoxia; MCT = monocrotaline; PMID = PubMed reference number; CKO = *Egln1*^*Tie2Cre*^; EH2 = *Egln1*^*Tie2Cre*^/*Hif2a*^*Tie2Cre*^; WT = wild type.

## Technical considerations for scRNAseq experiments

To optimize scRNAseq experiment design, it is important to select the appropriate platform based on research needs. Droplet-based platforms like 10x Genomics excel in high-throughput cell capture and are adept at detecting rare cell types, albeit with a typically lower number of genes detected per cell. In contrast, plate-based methods such as Smart-seq2 allow for full-length transcript sequencing, capturing more genes and offering insights into low abundance or alternatively spliced transcripts, though they capture fewer cells per sample. The decision to fix samples prior to scRNAseq hinges on factors such as the nature of the research questions, sample logistics, and the potential trade-off between RNA preservation and data quality [1, 2]. Fresh samples, free from the chemical alterations introduced by fixatives, are ideal for high-quality RNA extraction. However, fixation—utilizing methods like methanol or paraformaldehyde—can be necessary when immediate sample processing is impractical. The determination of cell number and sequencing depth should be informed by the complexity of the sample and the study’s objectives. More complex samples generally require analyzing a higher number of cells, and while lower sequencing depths can reveal cell population dynamics, more detailed expression analyses necessitate higher depths [3]. A minimum of 20,000 read pairs per cell is the recommended starting point for 10x Genomics Single Cell 3’ v3 and v4 platforms, though pilot studies and a review of related literature are advised for refining this estimate.

### Endothelial cells (artery)

Endothelial cell (EC) dysfunction is a key factor in PAH pathogenesis, initiating events that disrupt pulmonary vascular homeostasis and lead to pathological remodeling. Recent scRNAseq studies have shed light on EC biology in PAH. Saygin et al. [4] provided an initial glimpse into the EC transcriptome of human idiopathic PAH (IPAH) lung tissue through scRNAseq. While the small sample size (3 PAH lungs) limited statistical rigor for differentially expressed gene (DEG) and pathway analysis of EC and other cell clusters, an innovative analytic method employed by this study was SCENIC [5], a computational method for gene regulatory network reconstruction from scRNAseq data which links *cis*-regulatory sequences to single-cell gene expression to predict interactions between transcription factors (TF) and target genes. Their SCENIC analysis in ECs highlighted *SOX18* as a top candidate regulator of the IPAH EC transcriptome – it encodes a TF known to regulate endothelial barrier integrity and vessel development. ECs were analyzed as one cluster in this study. Similarly, a lung scRNAseq study by Thomas et al. [6] in the chronic hypoxic mouse model (4-week hypoxia; *n* = 1/group) also analyzed ECs as one cluster in which they identified a migratory and proliferative phenotype in response to hypoxia, consistent with prior observations. However, a challenge of analyzing lung ECs in scRNAseq is to subcluster ECs into those that originate from arteries, veins, or capillaries from either the pulmonary or systemic circulation. A comprehensive integrated lung EC atlas [7] is now available which distinguishes the various EC subtypes. Genes of interest can be queried and visualized on their webserver (http://www.lungendothelialcellatlas.com/) on either human or mouse UMAP (Uniform Manifold Approximation and Projection) plots. Furthermore, publicly available scRNAseq datasets (Table 1) can be reanalyzed and mapped onto a reference dataset such as the Human Lung Cell Atlas (HLCA) [8] which has resolved ECs into 8 subpopulations. The Seurat R package or the Azimuth web application (https://azimuth.hubmapconsortium.org/) can quickly transfer reference cell labels onto a query dataset at high resolution based on similarity of cell transcriptomes [9]. Another approach to circumvent the question of EC subtypes is to dissect out pulmonary arteries to enrich for pulmonary arterial ECs (PAECs) prior to scRNAseq. Asosingh et al. employed scRNAseq on cultured PAECs isolated from the main PA and branches from PAH and control human lungs (*n* = 3/group) [10]. Using a dimensionality reduction algorithm called TriMap [11] to visualize and cluster ECs in two dimensions, they identified 8 subclusters of PAECs and comparative analysis suggested a differential angiogenic and proliferative profile in PAH that may originate from a distinct subset of ECs. As the authors point out, whether these dysregulated EC subsets identified in cultured PAECs are also present in freshly isolated PAECs warrants follow up investigation. As was done in this study regarding dimensionality reduction, it could be useful to compare algorithms like TriMap which better preserves global structure of the data (i.e. similarity between different neighborhoods of cells) to more commonly used algorithms like t-SNE (t-distributed Stochastic Neighbor Embedding) and UMAP which better preserve local structure (i.e. cells within a neighborhood).

scRNAseq applied to animal models of PH can also elucidate endothelial heterogeneity, such as work by Hong et al. [12] where a focused analysis was performed on ECs derived from their earlier whole lung single-cell study [13] of two rat models of PH, Sugen-hypoxia (SuHx) and monocrotaline (MCT). The authors identified a dysregulated arterial endothelial cell subpopulation in PAH, termed EA2, characterized by the expression of *Tm4sf1*. This gene plays an important role in the regulation of cell development, growth, and motility and has been implicated in various cancers but never previously studied in PAH. This study utilized cell-cell communication and trajectory analysis methods to garner insights from the EA2 single-cell data, extending beyond mere differential gene expression and pathway analysis. CellPhoneDB [14], a tool for predicting ligand-receptor interactions between cell types, revealed increased CXCL12 signaling in the EA2 endothelial subpopulation within PH, indicating enhanced communication with other cell types. Additionally, CytoTRACE [15], which assesses cell differentiation states by analyzing the number of expressed genes per cell, suggested that EA2 cells were less differentiated compared to other endothelial subpopulations. The study also found that TM4SF1 is not only a marker for hematopoietic stem cells, but also co-expressed with hematopoietic marker CD45 in a subset of ECs suggesting that the EA2 subpopulation might possess stem/progenitor potential. In a preprint scRNAseq study of SuHx rat lungs at multiple time points [16], Cober and colleagues also identified *Tm4sf1* as a distinct marker of dysregulated arterial ECs. While a prior study has shown that *TM4SF1* knockdown in various cultured ECs causes significant impairment of various EC functions [17], in vivo manipulation of *Tm4sf1* in ECs will provide more insight into its role in PAH.

### Endothelial cells (capillary)

Capillary endothelial cells (ECs) are also a critical but understudied subtype implicated in the pathology of PAH [18]. For instance, the WHO categorizes pulmonary capillary hemangiomatosis (PCH), marked by abnormal proliferation of capillary ECs, as Group 1 PAH, sharing a similar hemodynamic phenotype with other subtypes within this group. On the other hand, loss of distal vessels and capillary rarefaction have also been described in PAH. Building off these observations, scRNAseq has enabled the investigation of capillary EC heterogeneity and its potential relevance to vascular remodeling in PAH. A study by Rodor et al. [19] performed scRNAseq on lungs and sorted ECs of EC lineage-traced SuHx mice and found upregulation of apoptotic, pro-migratory and pro-angiogenic genes specific to one of two subpopulation of capillary ECs which they call CapillaryB. The authors compared their mouse scRNAseq dataset with that of human by Saygin et al. and rat by Hong et al. and found overall cross-species concordance of EC subpopulations including CapillaryB. Two capillary subpopulations were first identified in human and mouse by Gillich et al. [20] which they termed alveolar capillary (aCap; aerocyte), specialized for gas exchange and trafficking of leukocytes, and general capillary (gCap), involved in capillary homeostasis and repair with stem/progenitor functions. With recent scRNAseq studies [7, 16, 21] increasingly adopting this terminology, it appears based on shared markers that Rodor et al.’s CapillaryB (marked by *Car4*) and CapillaryA (marked by *Sema3c*) correspond to aCap and gCap, respectively. By trajectory analysis with Slingshot [22], Rodor et al. also delineated changes in gene expression across the arteriovenous axis such as the PH-specific upregulation of the Serine/threonine-protein kinase *Sgk1* at the junction between capillaryA (gCap) and arterial ECs. *Sgk1* is known to regulate angiogenesis and its deficiency prevents hypoxia-induced PH in mice [23], suggesting it could be a key regulator of EC alterations. A recent preprint by Liu et al. [21] further implicates gCap ECs by employing lung scRNAseq and pseudotime trajectory analysis with Monocle 3[24] alongside capillary-specific EC genetic lineage tracing and spatial transcriptomics in the SuHx mouse model. Their findings suggest that gCap, but not aCap, gives rise to arterial ECs in the development of PH by SuHx. Single-cell spatial transcriptomics of human lung sections may shed more light on this model of EC transition in human PAH.

### Smooth muscle cells and pericytes

Vascular smooth muscle cells (SMCs) and pericytes, known as mural cells, envelop the endothelial layer of the pulmonary vasculature, and while they maintain vessel stability and function, their dysregulation may play an important role in the development of PAH [25]. In the human lung study, Saygin et al. noted IPAH upregulation of genes encoding specific ligands and their corresponding receptors between the EC cluster and the SMC/pericyte cluster suggesting altered cell-cell communication (*SLIT3*-*ROBO4*, *PDGFRB*-*PDGFB*, and *JAG1*-*NOTCH1*). Whether these interactions are specific to vascular SMCs, pericytes, or both requires further investigation as these cell types were part of one cluster in this study. In a detailed examination of vascular SMC heterogeneity, Crnkovic et al. [26] employed scRNAseq on pulmonary arteries (PAs < 5 mm diameter) isolated from human PAH lungs (and chronic hypoxic murine lungs) compared to control. The authors identified 4 major PASMC clusters which they found were conserved in human coronary arteries but less so in murine PAs by scRNAseq dataset integration using Seurat [27]. They categorized these clusters as contractile, oxygen sensing, synthetic, and fibroblast-like based on GO (Gene Ontology) enrichment analysis of cluster-enriched genes. While the authors did not find a loss or gain of a distinct SMC cluster in PAH as compared to healthy PAs, multiple analyses (in line with a previous proteomics analysis [28]) supported a shift from contractile to synthetic SMCs in PAH: trajectory inference with Monocle 3^24^ and RNA velocity analysis with scVelo [29], relative cell proportion analysis, ligand-receptor analysis (scTalk [30]), and differential gene expression analysis (Seurat’s *FindMarkers* function). Of note, unlike pseudotime algorithms, RNA velocity analysis with scVelo [29] can infer directionality of cell trajectories by leveraging the ratio of spliced and unspliced messenger RNA (mRNA) as a determinant of transcriptional dynamics. Furthermore, a preprint study by Wen et al. [31] performed scRNAseq on human PAs from PAH compared to control and also found SMC phenotypic switching in PAH (from contractile to fibroblast-like SMCs). Their data suggested M1 macrophage polarization as a regulator of this switch. Crnkovic et al. and Wen et al. did not identify a distinct pericyte cluster, possibly due to profiling of more proximal PAs where pericytes may be less abundant compared to distal microvasculature (i.e. small precapillary arteries < 30 μm in diameter) [32]. Similarly, Saygin et al. did not distinguish a pericyte cluster in whole lung. Adopting a single-nucleus approach or increasing the sample size may improve the yield of this cell type in future PAH single-cell studies, as demonstrated in the recent Human Lung Cell Atlas [8]. Interestingly, mural cells from the Saygin et al. dataset were re-clustered in a recent study by Kim et al. [33] uncovering a small but distinct pericyte population. Further investigation by Kim et al. demonstrated that pericytes in both human PAH patients and mice exposed to chronic hypoxia (3 weeks) exhibit elevated levels of HIF2α. Notably, they found that overexpressing HIF2α specifically in pericytes aggravated PH in the chronic hypoxia model, underscoring a potential mechanistic link in PAH pathology.

### Fibroblasts

Beyond endothelial and mural cells, fibroblasts and myofibroblasts are also important vascular cells in PAH pathobiology [18], although their exact contributions remain to be fully elucidated. In the Sayin et al. study, analysis of the lung fibroblast cluster in PAH suggested activation of pathways commonly dysregulated in the disease, such as WNT and TGF-beta signaling, although the statistical significance was constrained by a small sample size. Further investigation is needed to determine the specific contributions of adventitial fibroblasts and myofibroblasts within this cluster. Crnkovic et al.’s study on PAs noted that their (adventitial) fibroblast cluster was homogenous without clear subpopulations. Their analysis suggested that the fibroblast-like SMC subpopulation could have originated from both fibroblasts and SMCs based on trajectory analysis. Although this study did not specifically identify myofibroblasts, further investigation could help determine how the identified fibroblast-like SMC subpopulation relates to myofibroblasts. Wen et al.’s preprint on PAs also identified fibroblast-like SMCs, which were distinct from myofibroblasts in their dataset. Overall, Wen et al. described 8 fibroblast subclusters and 2 myofibroblast clusters each with unique functions as suggested by pathway analysis, from lipid to bone metabolism. It remains to be determined whether these subpopulations have specific roles in PAH or exhibit distinct transcriptional profiles in independent scRNAseq or spatial transcriptomics datasets.

### Monocytes

In addition to vascular cells, immune cells are also key players in PAH pathogenesis, with scRNAseq offering an unbiased approach to analyzing and prioritizing various immune populations within the lung. In the earlier work by Hong et al. which applied lung scRNAseq to the SuHx and MCT rat models, comparative analysis across lung cell types and both models was conducted using multiple approaches: a Euclidean distance-based approach to assess global transcriptomic shifts [34], enrichment analyses for known pathways and PAH genes using FGSEA [35], and GWAS (genome-wide association study) enrichment analysis using Mergeomics [36].

These analyses prioritized SuHx non-classical monocytes and its NFkB signaling as particularly relevant to human PAH. Pharmacotranscriptomic analysis was also performed using Connectivity Map [37] which uses a pattern matching algorithm and a drug transcriptomic database to predict drugs that might reverse or mimic a query signature. This analysis identified Treprostinil, one of the most effective PAH therapies in clinical use, as the top drug predicted to reverse the SuHx non-classical monocyte signature out of > 2400 drugs screened. These findings build off prior research on non-classical monocytes [38, 39] to further underscore the critical role non-classical monocytes might play in PAH pathogenesis. This early lung scRNAseq study retrieved fewer vascular cells than expected, highlighting a possible shortfall in tissue dissociation protocols to comprehensively recover diverse cell populations in complex tissues. As we will discuss later in this review, emerging methods like single-nucleus RNA sequencing may overcome these challenges.

### Neutrophils

Apart from cells residing in the lung, circulating immune cells like neutrophils in the peripheral blood may also play a crucial role in PAH. Zhang et al. [40] performed scRNAseq on peripheral immune cells of IPAH vs. control (*n* = 3/group) patients with a focus on neutrophil heterogeneity. They employed a number of methods to further characterize the 5 neutrophil subclusters they identified such as trajectory analysis with Monocle 2[41], cell-cell communication analysis with CellPhoneDB, and weighted gene coexpression network analysis (WGCNA) [42]. They identified PAH DEGs specific to each neutrophil subcluster with different functional annotations ranging from antigen presentation to natural killer-mediated cytotoxicity. Notably, they demonstrated by flow cytometry in an independent cohort of PAH patients (*n* = 75) that one of the neutrophil subpopulations, characterized by high expression of MMP9, was associated with worse survival. Neutrophil-derived MMP9 is known to promote angiogenesis such as in cancer [43] but its precise role in the dysregulated angiogenesis and vascular remodeling of PAH will require further study. While this study by Zhang et al. centered on neutrophils, future research could expand to include analysis of other immune cell populations in circulation, such as regulatory T cells and CD8 + T cells, which are altered in PAH patients [44, 45] but not yet systematically studied by scRNAseq.

### Challenges and future directions

The advent of scRNAseq technology has revolutionized our investigation of PAH at the single-cell level, significantly enriching our understanding of its pathobiology. Despite its advancements, scRNAseq in PAH research encounters challenges such as scarce fresh human tissue samples relevant to PAH, loss of cells’ spatial context in tissue architecture, and biases in cell composition and gene expression changes due to the stress of tissue dissociation. The enzymatic digestion required to create single-cell suspensions can also limit the recovery of delicate or matrix-embedded cell types. Emerging techniques like single-nucleus RNAseq (snRNAseq) are promising in addressing these challenges, especially in complex tissues like the lung and right ventricle. They have been shown to detect genes comparably to scRNAseq and can be performed on frozen tissue, which allows for the analysis of multiple archived samples while reducing technical variability [46, 47]. Sample multiplexing and fixed RNA profiling, utilizing technologies such as 10x Genomics or MULTI-seq (implemented in Cober et al.’s preprint), improve throughput and cost-efficiency, facilitating the analysis of more samples simultaneously.

Integrating scRNAseq with other single-cell technologies like ATAC sequencing (scATACseq) can provide a multi-dimensional view of the broader molecular landscape. While scRNAseq excels in RNA-level cell profiling, scATACseq unveils the chromatin accessibility landscape, shedding light on the regulatory mechanisms influencing gene expression patterns identified through scRNAseq. This integration facilitates a comprehensive understanding of the transcriptomic and epigenomic interplay, crucial for deciphering complex gene regulatory networks and cellular dynamics. Furthermore, emerging fields like single-cell proteomics and metabolomics are enriching our knowledge by revealing protein expression levels, post-translational modifications, and active metabolic pathways in individual cells. These insights bridge the gap between transcriptomic data and cellular functionality, providing a clearer picture of the cellular machinery in action. Moreover, spatial transcriptomics technologies such as Nanostring’s CosMx and 10x Genomics’ Visium HD enable the precise mapping of gene expression at subcellular resolution within the native tissue architecture and can offer detailed views of cellular interactions and heterogeneity in specific spatial contexts, such as the remodeled vessels of PAH-affected lungs. The integration of these diverse single-cell data types will be instrumental in constructing a multi-layered holistic understanding of dysregulated biological processes and disease mechanisms, offering unparalleled resolution in studies of PAH and beyond.

Analytical methods for single-cell data are also evolving, becoming more rigorous and sophisticated. Differential gene expression analysis in scRNAseq studies commonly use methods like the Wilcoxon rank-sum test but fail to consider the correlation between cells from the same individual, leading to potential biases [48, 49]. The current best practice, likely to influence future PAH single-cell analyses, involves aggregating gene counts by sample to create ‘pseudobulks,’ then analyzing them with statistical packages designed for bulk expression data, such as edgeR [50], DEseq2[51], and limma [49], to account for sample-level variations. Cell-cell communication inference methods often depend on databases of ligands and receptors, which may be biased toward certain biological pathways and tissues [52]. The choice of both the inference method and the database can significantly affect the predictions of interactions. LIANA [52] is notable for its ability to provide a consensus ranking of predictions across various methods and databases [49]. While these tools typically utilize gene expression data from scRNAseq, integrating spatial and/or proteomic data promises to enhance our understanding of cell-cell interactions, delineating communication niches and identifying key targets in disrupted communication networks of PAH.

As scRNAseq becomes more accessible, its deployment in PAH research demands careful consideration. Studies leveraging this technology ought to be grounded in specific research questions to generate meaningful and interpretable data that shed light on the intricate biological processes and mechanisms at play. Moreover, findings from scRNAseq need validation through techniques such as immunofluorescence to confirm the protein-level presence and functional relevance of the identified transcripts within their tissue context. The use of scRNAseq on human PAH tissues necessitates meticulous attention to factors like sample quality, disease stage, anatomic specifics, and confounders such as age, sex, and medications. Typically, samples are derived from patients with advanced-stage PAH, which could obscure the discovery of genes and pathways implicated in the disease’s onset or progression. Additionally, a focus on proximal pulmonary arteries may not capture the most relevant PAH pathobiology occurring in the remodeled vessels of the distal lung. Furthermore, ensuring consistent and accurate interpretations requires a careful evaluation of the variability in cellular markers and subtypes reported across various studies and animal models. These considerations are equally critical when re-analyzing publicly available scRNAseq datasets. When integrating single-cell data from various studies or platforms, employing rigorous batch correction methods—such as Harmony [53] or canonical correlation analysis [54]—is essential to accurately interpret the data and discern true biological signals [49]. Moreover, providing open access to single-cell datasets to the wider research community is crucial, as it not only reinforces transparency and reproducibility but also serves as a catalyst for scientific innovation, thereby expanding our understanding of this complex disease.

## Conclusion

In this review, we have showcased a series of studies that have propelled a new era of discovery in the cellular dynamics of PAH using scRNAseq, spanning different species and tissues. These investigations have underscored the power of scRNAseq to identify novel molecular targets, decipher cellular heterogeneity, and confirm the vital roles of both vascular and immune cells in PAH. While considerable advances have been made, there remains ample scope to exploit the ever-advancing single-cell technologies to further delineate PAH’s cellular underpinnings, ranging from single-cell spatial profiling to integrative omics analyses. As we deepen our molecular grasp of PAH through single-cell multiomics, we simultaneously broaden the horizon for developing innovative therapeutic strategies for patients.

## Data Availability

No datasets were generated or analysed during the current study.
